# Detection of *Echinococcus multilocularis* in Carnivores in Razavi Khorasan Province, Iran Using Mitochondrial DNA

**DOI:** 10.1371/journal.pntd.0001379

**Published:** 2011-11-22

**Authors:** Molouk Beiromvand, Lame Akhlaghi, Seyed Hossein Fattahi Massom, Iraj Mobedi, Ahmad Reza Meamar, Hormozd Oormazdi, Abbas Motevalian, Elham Razmjou

**Affiliations:** 1 Department of Parasitology and Mycology, School of Medicine, Tehran University of Medical Sciences, Tehran, Iran; 2 Department of Thoracic Surgery, Ghaem Educational, Research and Treatment Center, Mashhad University of Medical Sciences, Mashhad, Iran; 3 Department of Medical Parasitology and Mycology, School of Public Health, Tehran University of Medical Sciences, Tehran, Iran; 4 Department of Epidemiology and Biostatistics, School of Public Health, Tehran University of Medical Sciences, Tehran, Iran; Universidad Nacional Autónoma de México, Mexico

## Abstract

**Background:**

*Echinococcus multilocularis* is the source of alveolar echinococcosis, a potentially fatal zoonotic disease. This investigation assessed the presence of *E. multilocularis* infection in definitive hosts in the Chenaran region of Razavi Khorasan Province, northeastern Iran.

**Methodology/Principal Findings:**

Fecal samples from 77 domestic and stray dogs and 14 wild carnivores were examined using the flotation/sieving method followed by multiplex PCR of mitochondrial genes. The intestinal scraping technique (IST) and the sedimentation and counting technique (SCT) revealed adult *Echinococcus* in the intestines of five of 10 jackals and of the single wolf examined. Three jackals were infected only with *E. multilocularis* but two, and the wolf, were infected with both *E. multilocularis* and *E. granulosus*. Multiplex PCR revealed *E. multilocularis*, *E. granulosus*, and *Taenia* spp. in 19, 24, and 28 fecal samples, respectively. *Echinococcus multilocularis* infection was detected in the feces of all wild carnivores sampled including nine jackals, three foxes, one wolf, one hyena, and five dogs (6.5%). *Echinococcus granulosus* was found in the fecal samples of 16.9% of dogs, 66.7% of jackals, and all of the foxes, the wolf, and the hyena. The feces of 16 (21.8%) dogs, 7 of 9 (77.8%) jackals, and all three foxes, one wolf and one hyena were infected with Taenia spp.

**Conclusions/Significance:**

The prevalence of *E. multilocularis* in wild carnivores of rural areas of the Chenaran region is high, indicating that the life cycle is being maintained in northeastern Iran with the red fox, jackal, wolf, hyena, and dog as definitive hosts.

## Author Summary

*Echinococcus multilocularis* causes alveolar echinococcosis, a serious zoonotic disease present in many areas of the world. The parasite is maintained in nature through a life cycle in which adult worms in the intestine of carnivores transmit infection to small mammals, predominantly rodents, via eggs in the feces. Humans may accidentally ingest eggs of *E. multilocularis* through contact with the definitive host or by direct ingestion of contaminated water or foods, causing development of a multivesicular cyst in the viscera, especially liver and lung. We found adult *E. multilocularis* in the intestine and/or eggs in feces of all wild carnivores examined and in some stray and domestic dogs in villages of Chenaran region, northeastern Iran. The life cycle of *E. multilocularis* is being maintained in this area by wild carnivores, and the local population and visitors are at risk of infection with alveolar echinococcosis. Intensive health initiatives for control of the parasite and diagnosis of this potentially fatal disease in humans, in this area of Iran, are needed.

## Introduction

*Echinococcus multilocularis* is the agent of alveolar echinococcosis, a potentially fatal zoonotic disease [1], [2]. The life cycle of *E. multilocularis* is sylvatic; adult worms are found in wild carnivores, principally foxes, and in the raccoon dog, wolf, coyote, and jackal, while their metacestodes develop in small mammals, predominantly rodents such as Cricetidae, Arvicolidae, and Muridae [3], [4], [5], [6], [7]. In some rural areas domestic dogs and sometimes cats can be definitive hosts after acquiring the infection from wild rodents, and thus become a major zoonotic risk for infecting humans [3], [8], [9]. Humans can serve as an aberrant intermediate host for *E. multilocularis*, with transmission occurring through direct contact with the definitive host or by ingestion of contaminated water, vegetables, or other foods [10]. Human alveolar echinococcosis is a lethal zoonotic disease caused by infection with the multivesiculated metacestode of *E. multilocularis* [11], [12].

The geographical distribution of the parasite is restricted to the northern hemisphere. The cestode has been reported in areas of central Europe, the Near East, Russia, central Asian republics, northern Japan, and Alaska [11], [13], [14], [15]. In the Middle East, cystic echinococcosis is prevalent in most countries, although a low prevalence of alveolar echinococcosis is reported in Iran, Iraq, and Tunisia [16]. In Asia, canine infections have been recorded in dogs in China, Kazakhstan and Kyrgyzstan [17], [18], [19]. *Echinococcus multilocularis* in canids was reported in northwestern Iran for the first time in 1971 [20], [21]. Further investigation in 1992 found its infection in 22.9% of red foxes (*Vulpes vulpes*) and 16% of jackals (*Canis aureus*) [22]. The latest research in 2009 reported no evidence of *E. multilocularis* infection in canids of the Moghan Plain in northwest Iran [23]. The majority of previous research focused only on the northwestern part of the country [20], [21], [22], [23]. Alveolar echinococcosis, based on histopathological and clinical data, was first reported in a village in Chenaran County of Razavi Khorasan Province in 2007 [24]. The disease was subsequently confirmed by molecular evaluation, and a second case reported (E. Razmjou, unpublished data). Razavi Khorasan Province is located in northeastern Iran, near the border with Turkmenistan, where *E. multilocularis* is endemic. A pilot study revealed that suitable hosts such as foxes, jackals, dogs, wolves, and rodents are frequently present near villages in the mountains of Chenaran region. The presence in Razavi Khorasan of suitable conditions for completing the life cycle of this parasite, such as presence of definitive and intermediate hosts in mountainous areas and proximity to other countries where the parasite is endemic, led to the present study to assess the prevalence of *E. multilocularis* infection in carnivores, and to identify natural definitive hosts of this life-threatening parasite in the Chenaran region of Razavi Khorasan Province, Iran.

## Materials and Methods

### Ethics statement

Animals were shot under license from the Iran Environment Protection Organization, solely for the purpose of investigating the presence of *Echinococcus multilocularis* in wild carnivores. The Protocol of this investigation was reviewed and approved by the Ethics Committee of Tehran University of Medical Sciences.

### Study area

**Figure 1:**
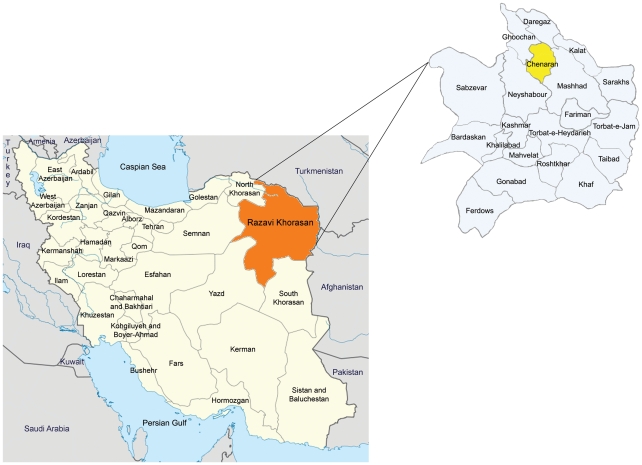
Map of Iran. Razavi Khorasan Province is indicated by orange. The study area, Chenaran County, is highlighted with yellow. Based on the map of Razavi Khorasan Province at http://en.wikipedia.org/wiki/File:Locator_map_Iran_Razavi_Khorasan_Province.png by Uwe Dering, highlighted by Dr. Blofeld.

The study area, the Chenaran region, covers approximately 2400 km^2^ in northeastern Iran, 55 kilometers northwest of Mashhad (Figure 1) (36°4′N, 59°7′E). It lies between the Binalood Heights and the Hezar Masjed Mountains. Chenaran city is surrounded by rural areas, mainly consisting of human habitations, gardens, farms, and moorland. The region has cold and snowy winters and mild summers. The average annual temperature is 13.4°C with variable rainfall; mean annual precipitation of 212.6 mm. It is rich in wildlife, including carnivores and small rodents appropriate for supporting the life cycle of *E. multilocularis*.

### Sample collection

From November 2009 to January 2010, fecal samples from 77domestic and stray dogs from 17 villages and the entire gut of three foxes, ten jackals, and one wolf (*Canis lupus pallipes*), either shot or killed accidentally, were collected. In addition, during October and November 2010, the intestines of one fox and one hyena (*Hyena hyena*) killed on roads were added to our samples. A standard form including place and date of killing was completed. Fecal samples were collected from the rectum of each wild carnivore. The intestine and fecal samples were placed in labeled ziploc bags, stored at −80°C for at least seven days [25] to reduce the risk of laboratory infection by inactivating any *Echinococcus* oncospheres and other infective materials, and subsequently stored at −20°C until further examination.

### The intestinal scraping technique (IST)

The intestinal scraping technique was performed as described by Deplazes and Eckert [25]. The intestine was opened full length and after removal of undigested food and visible parasites from the proximal, middle, and posterior parts of the small and large intestine, 15 deep mucosal scrapings were taken using microscope slides. Material adhering to the slides was transferred to plastic Petri dishes and examined stereomicroscopically at 120× magnification. *Echinococcus* worms were isolated and stored in 85% ethanol for molecular examination and in 10% formalin for morphological diagnosis.

### The sedimentation and counting technique (SCT)

The sedimentation and counting technique was done as previously described [26]. The intestine was cut into 10 cm pieces and each was placed in a flask containing one liter of 0.9% saline. After vigorous shaking for a few seconds, the pieces of intestine were pressed firmly between the fingers using gloves and with care to avoid contamination with eggs to remove attached worms. The supernatant was decanted and the procedure was repeated several times with physiological saline. The sediment was placed in plastic Petri dishes and examined stereomicroscopically at 120× magnification. All isolated worms, including *Echinococcus*, were stored in 85% ethanol and 10% formalin for molecular and microscopic identification, respectively.

### Taeniid egg isolation with zinc chloride flotation

Fecal samples were submitted to flotation with zinc chloride for isolating parasite eggs. Each sample (4–5 g) was stirred into 50 ml distilled water until completely dispersed. The suspension was passed through four layers of gauze and large particles removed. The suspension was transferred into a 50 ml Falcon tube and centrifuged at 1000×g for 5 min. For isolating eggs, zinc chloride solution (specific gravity 1.45 g ml^−1^) was added to sediment up to a final volume of 12 ml and, after complete mixing, centrifuged at 1000×g for 30 min [27]. The supernatant was passed through sequential sieves on 50 ml falcon tubes with metal and polystyrene screens of mesh sizes 37 and 20 µm, respectively [27]. The sieves were inverted and washed thoroughly with distilled water containing 0.2% Tween 20. After adding phosphate-buffered saline (PBS; pH 7.2) to a final volume of 50 ml, suspensions were centrifuged at 1000×g for 30 min, the supernatant fraction was aspirated, and sediment (approximately 400 µl) was transferred to 1.5 ml tubes and stored at −20°C until further examination.

### Morphological examination

Detection of *Echinococcus* spp. was based on morphological characteristics. Adult worms of *E. multilocularis* and *E. granulosus* were differentiated using morphological characteristics including size, length of gravid proglottids, shape of the uterus, number of eggs per proglottid, and position of genital pore after acetic acid alum carmine staining and mounting in Canada balsam.

### DNA extraction

DNA of adult worms and taeniid eggs was extracted using the QIAamp DNA Mini kit (Qiagen, Germany) according to the protocol of Verweij et al. [28] with slight modifications. Briefly, one *Echinococcus* worm was removed from 85% ethanol and washed in sterile PBS buffer three times. The worm was then placed in 200 µl of PBS buffer and, after 10 min boiling at 100°C, an equal volume of ATL buffer plus 10% proteinase K was added and completely mixed and incubated two hours at 55°C in heat block. DNA extraction was continued according to manufacturer's instructions with the minor modification of increasing incubation time to five minutes to increase the yield of DNA in the final step. DNA was stored at −20°C until analysis. Before submitting the eggs from fecal samples to the DNA extraction procedure described for adult worms, they were subjected to seven freeze/thaw cycles, using liquid nitrogen and boiling water, to disrupt the egg wall. Then, 200 µl of the sample was heated at 100°C for 10 min as in Verweij's procedure [28]. The concentration of extracted DNA was measured spectrophotometrically by Biophotometer (Biophotometer Plus, Eppendorf, Germany).

### Multiplex PCR

Multiplex PCR of adult worms and eggs was performed as described [29]. The mitochondrial multiplex reaction was designed to amplify a 395 bp fragment of NADH dehydrogenase subunit 1 (*nad1*) of *E. multilocularis* and 117 bp and 267 bp of a small subunit of ribosomal RNA (*rrnS*) of *E. granulosus* and other *Taenia* spp., respectively. Primers, conditions, and parameters for PCR were as previously described [29]. All samples were tested in 25 µl amplification reaction mixtures with 12.5 µl of the master mix (QIAGEN Multiplex PCR, Germany), 2.5 µl of primers (2 µM of primers Cest1, Cest2, Cest3, Cest4 and 16 µM of primer Cest5 in H_2_O), 8 µl H_2_O, and 2 µl of template DNA. Initially, multiplex PCR was confirmed with standard DNA of *E. multilocularis*, *E. granulosus*, *Taenia multiceps*, and *T. hydatigena* provided by Professor Deplazes of the Institute of Parasitology of Zurich, Switzerland. Additionally, for all PCR reactions one negative control without DNA and one positive control with standard DNA were included to confirm the results of multiplex PCR. Finally, 10 µl of the PCR products were loaded on 2% (W/V) agarose gels, and stained with ethidium bromide to visualize by electrophoresis.

The results of multiplex PCR were confirmed by single PCR using the primer pair Cest1/Cest2 and Cest4/Cest5 for *E. multilocularis and E. granulosus*, respectively [29], and EM-H15/EM-H17 [9] for *E. multilocularis*.

All *E. multilocularis* PCR-positive samples were confirmed by sequencing of a 395 bp amplified fragment. PCR products were excised from agarose gels and purified using the QIAquick Gel Extraction Kit (QIAgen, Germany), according to the manufacturer's instructions. Products were sequenced in both directions using the Cest1/Cest2 primers by MilleGen Company (France). Sequences were read by CHROMAS (Technelysium Pty Ltd., Queensland, Australia) and aligned using the DNASIS MAX (version 2.09; Hitachi, Yokohama, Japan) software program.

## Results

### IST, SCT and morphology

The entire small intestine of 16 wild carnivores comprising 10 jackals, four foxes, one hyena, and one wolf were examined by both IST and SCT. These techniques found 6 of 16 (37.5%; 95% CI: 18.5%–61.4%) wild canids to be infected with *Echinococcus* spp. The worms were isolated from five of 10 (50%, 95% CI: 23.7–76.3%) jackals and one wolf, while the remaining jackals, the foxes, and the hyena tested negative. The intensity of infection was classified as low (1–100), medium (101–1000), or high (>1000) worm burden [30]. All positive jackals showed a high *Echinococcus* worm burden, while the wolf had a low burden.

**Figure 2:**
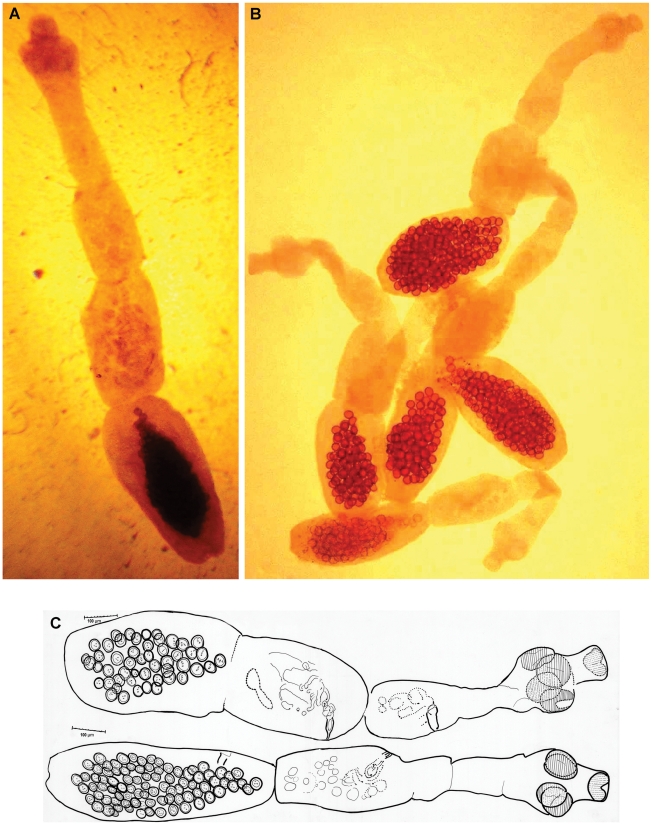
*Echinococcus multilocularis* isolated from a jackal in Razavi Khorasan Province. A) in normal saline solution (×100); B) with acetic acid alum carmine staining (×40); C) Drawings of *E. multilocularis* (×400) (made with the aid of Camera Lucida).

We differentiated all *Echinococcus* worms by microscopic examination (Figure 2). Among six *Echinococcus* positive samples, three jackals (30%) had a single species infection with *E. multilocularis* but two jackals (20%) and the wolf were infected with both *E. multilocularis* and *E. granulosus*.

### Flotation technique

To detect eggs, fecal samples of dogs were investigated by direct microscopic examination and the flotation method. Eggs were observed in 13 of 77 (16.9%, 95% CI: 10.1–26.8%) dog fecal samples.

### Molecular analysis

**Figure 3:**
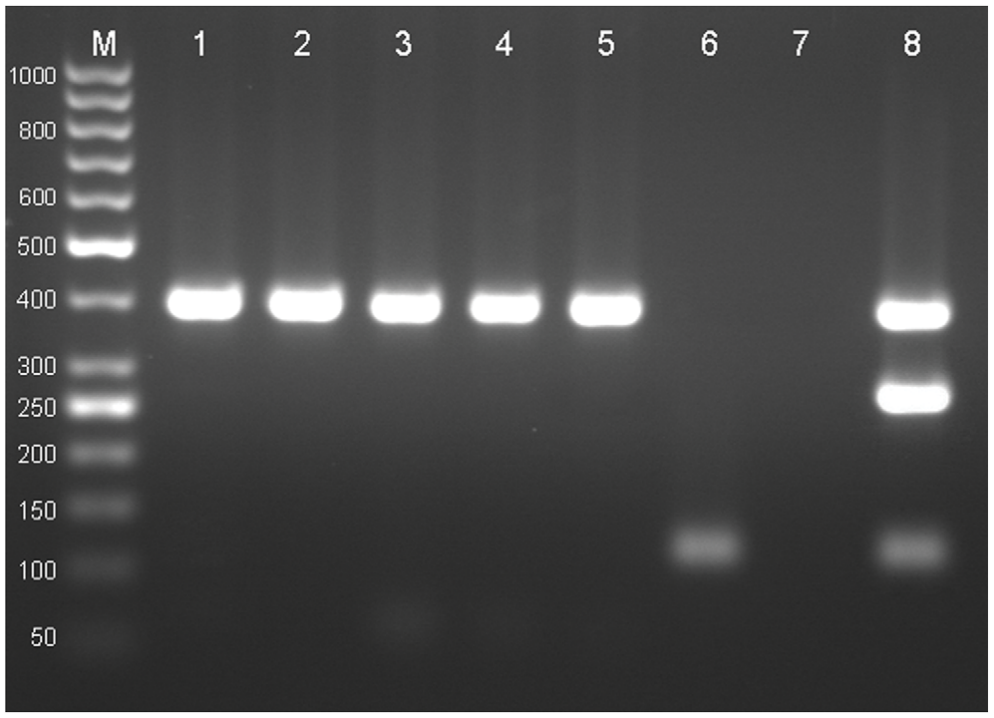
Multiplex PCR amplification of DNA extracted from adult worms in Razavi Khorasan Province. Lane M, 50 bp DNA ladder (Fermentas; Cat No SM0373); Lane 1–5, *Echinococcus multilocularis* taken from five jackals; Lane 6 *E. granulosus* isolated from a wolf; Lane 7, negative control; Lane 8, positive control, a mixture of standard DNA of *E. multilocularis*, *E. granulosus*, and *Taenia. hydatigena*.

**Figure 4:**
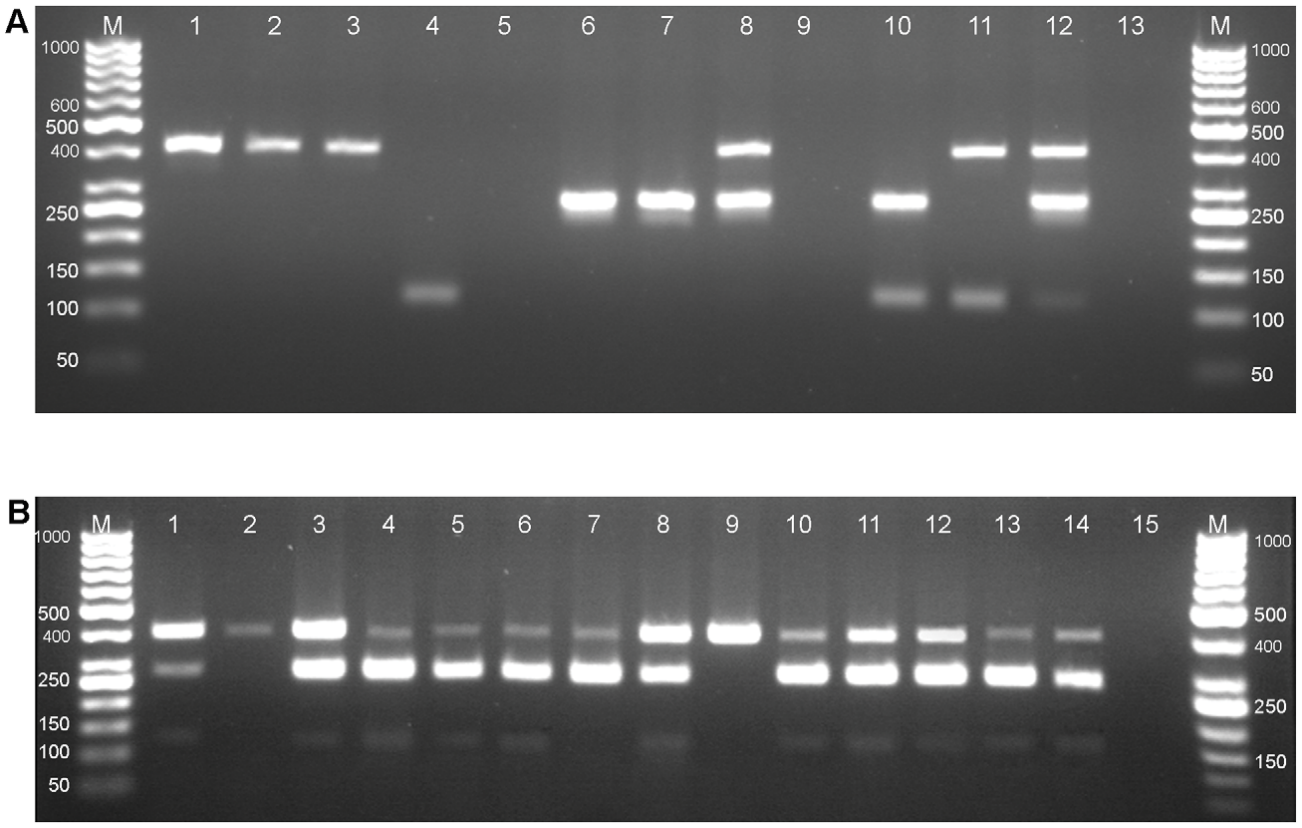
Multiplex PCR amplification of DNA extracted from fecal samples. A. of dogs: Lane M; 50 bp ladder, Lane 1, positive control; Lane 2–11 samples; Lane 13, negative control. B. of wild carnivores: Lane M, 50 bp ladder, lane 1–2 and 6–12, jackal samples; Lane 3–5, fox samples; Lane 13, wolf sample; Lane 14, hyena sample; Lane 15, negative control.

The result of multiplex PCR by amplification of 395 bp fragment of *nad1* indicated that 19 of the carnivores were infected with *E. multilocularis*. The 117 bp fragment of *rrnS* identified *E. granulosus* in 24, and the 267 bp fragment found *Taenia* spp. in 28 of the fecal samples. *Echinococcus multilocularis* infection was detected in the feces of all wild carnivores (100%; 95% CI: 78.5–100%) including nine jackals, three foxes, one wolf and one hyena, and five dogs (6.5%; 95% CI: 2.8–14.3%). *Echinococcus granulosus* was found in the fecal sample of 16.9% (95% CI: 10.1–26.8%) of dogs, 66.7% (95% CI: 35.4–88.0%) of jackals, and all of the foxes, the wolf, and the hyena. The feces of 16 of 77 (21.8%; 95% CI: 13.2–31.1%) dogs, 7 of 9 (77.8%; 95% CI: 45.3–94.0%) jackals, and all three foxes, one wolf and one hyena were infected with *Taenia* spp. (Table 1) (Figures 3, 4).

**Table 1:** Prevalence of *E. multilocularis*, *E. granulosus*, and *Taenia* spp. infection in fecal samples of canids.

Host^a^	*E. multilocularis* (%)	*E. granulosus* (%)	*Taenia* spp. (%)
Dog	5 (6.5%)	13 (16.9%)	16 (21.8%)
Jackal	9 (100%)	6 (66.7%)	7 (77.8%)
Fox	3 (100%)	3 (100%)	3 (100%)
Hyena	1 (100%)	1 (100%)	1 (100%)
Wolf	1 (100%)	1 (100%)	1 (100%)
Total	19	24	28

a Canids host: 77 dogs, nine jackals, three foxes, one hyena, and one wolf from Razavi Khorasan Province.

Among 26 PCR positive dog samples, a single DNA fragment amplified in 18 samples indicated two *E. multilocularis* (2.6%; 95% CI: 0.7–9.0%), six *E. granulosus* (7.8%; 95% CI: 3.6–16.0%), and ten *Taenia* spp.(13%; 95% CI: 7.2–22.3%) infected dogs. Two species-specific fragments in seven cases showed five dogs (6.5%; 95% CI: 2.8–14.3%) to be co-infected with *E. granulosus* and *Taenia* spp., and two *E. multilocularis* infected dogs were also infected with *Taenia* spp. (1.3%; 95% CI: 0.2–7.0%) or *E. granulosus* (1.3%; 95% CI: 0.2–7.0%). Three amplicons revealed one dog (1.3%; 95% CI: 0.2–7.0%) infected simultaneously with *Taenia* spp. and two species of *Echinococcus*. Of 14 *E. multilocularis* infections in wild carnivorous, 11 showed triple infections including six jackals (66.7%), three foxes (100%), the hyena and the wolf. One jackal was co-infected with *Taenia* spp., and two jackals were infected with *E. multilocularis* (Table 2; Figure 4). The prevalence of *E. multilocularis* infection was high in wild carnivores (100%; 95% CI: 78.5–100%), whereas the rate of infection in domestic and stray dogs was low (6.5%; 95% CI: 2.8–14.3%). The results of multiplex PCR were confirmed with single PCR. Sequencing results of amplicons obtained from worms and eggs were identified as *E. multilocularis*. The nucleotide sequences of the amplified *nad1* were equivalent to positions 7645 to 8040 of the published *E. multilocularis* mitochondrion complete genome (accession no. AB018440). Sequences were aligned using DNASIS MAX (version 2.09; Hitachi, Yokohama, Japan) with the published reference sequences. Analysis revealed 100% identity between our isolates and the corresponding published reference sequences for *E. multilocularis*. The nucleotide sequence data reported in this paper will appear in the DDBJ/EMBL/GenBank nucleotide sequence databases with the accession numbers AB617846–AB617855 and AB621793–AB621801.

**Table 2:** Co-infections with *E. multilocularis*, *E. granulosus* and *Taenia* spp in fecal samples of canids.

Host	No	Em	Eg	T	Em/T	Eg/T	Em/Eg	Em/Eg/T
Dog	77	2 (2.6%)	6 (7.8%)	10 (13.0%)	1 (1.3%)	5 (6.5%)	1 (1.3%)	1 (1.3%)
Jackal	9	2 (22.2%)	0	0	1 (11.1%)	0	0	6 (66.7%)
Fox	3	0	0	0	0	0	0	3 (100%)
Hyena	1	0	0	0	0	0	0	1 (100%)
Wolf	1	0	0	0	0	0	0	1 (100%)

Em: *E. multilocularis*; Eg: *E. granulosus*; T: *Taenia* spp.

## Discussion

Iran is located in the Middle East and central Eurasia. It is bordered on the north by Armenia, Azerbaijan, Turkmenistan and Caspian Sea. Afghanistan and Pakistan are Iran's direct neighbors in the east. The country borders the Persian Gulf and the Gulf of Oman in the south, Iraq in the west, and Turkey to the north-west (Figure 1).

In the Middle East, echinococcosis is one of the most important zoonotic diseases [16]. Cystic echinococcosis (CE) and alveolar echinococcosis (AE) have been reported in the Mediterranean region, but CE is more prevalent [31], [32]. Although Iran is an endemic area for echinococcosis, most studies have been carried out only on *E. granulosus*. Mobedi and Sadighian in 1971 reported *E. multilocularis* for the first time in three of 30 red foxes tested from northwest Ardebil Province [20], [21]. A study of *E. multilocularis* in carnivores of that area was followed in 1992 by Zariffard and Massoud [22] showing comparable results, infection in 22.9% (16/70) of red foxes and 16% (4/25) of the jackals. In a study in 2009 Zare-Bidaki et al. did not observe *E. multilocularis* in the investigated canids [23]. With the exception of these three studies, *E. multilocularis* infection has not been investigated outside of the northwest part of the country. Although there is limited information about AE in Iran, Torgerson et al. [10] suggested that Iran, since it is bordered by highly endemic countries, is an endemic area for *E. multilocularis*, and that the estimated annual incidence of eleven cases of AE are likely underreported. Molecular confirmation of some AE reports from inhabitants of a village in Chenaran County of northern Razavi Khorasan Province [24] (E. Razmjou, unpublished data), which neighbors a hyperendemic country, Turkmenistan, led to our investigation of the establishment of *E. multilocularis'* life cycle in this area.

Assessment of the occurrence of *E. multilocularis* in definitive hosts showed that this cestode has a high prevalence in the wild carnivores of the Chenaran area in northeastern Iran (100%; 95% CI: 78.5–100%). In comparison to the prevalence of infection in foxes in Belgium (24.55%) [33], Switzerland (47%–67%) [26], Ukraine (36%) [34], Kyrgyzstan (64%) [35], and Japan (49%) [36], it is assumed that Razavi Khorasan Province is hyperendemic for this tapeworm.

The significantly lower prevalence of *E. multilocularis* infection in dogs (6.5%) than in other carnivores (100%) may be due to decreased ingestion of metacestode infected intermediate hosts through controlled and limited diet of domestic dogs. As the multiplex PCR is subject to amplification of DNA from taeniid eggs, the results could also be associated with overlooking some positive cases due to prepatent infections, the intermittent egg excretion during the patent period [29], or degradation of taeniid eggs in the environment and unsuitable conditions such as solar radiation [37].

The rate of infected dogs in our study (6.5%) was less than reported in China (12%) [38] and Kyrgyzstan (18%) [19]. On the other hand, the infection rate observed in our study was considerably greater than prevalence reported in Germany (0.24%) [4] and Lithuania (0.8) [39]. The low rates found there may have been a consequence of conducting PCR only on positive taeniid egg samples by the sieving/flotation method. Our experiment showed *Taenia* eggs in only 16.9% of the 77 dog feces by sieving/flotation and microscopic examination. This increased to 33.9% with multiplex PCR on the flotation material. It assumed that results might be related to difficulty in detecting small numbers of eggs by microscopic examination, while DNA of a single taeniid egg from a sieved fecal sample can be amplified by the multiplex PCR [29].

While the prevalence of infection in dogs is low, the large population of domestic and stray dogs in the villages that are in close contact with inhabitants must be considered a potential source and risk factor for transmission of *E. multilocularis* to humans. The results of multiplex PCR in the current study showed that most of the wild (78.6%) and some domestic (2.6%) canids were co-infected with *E. granulosus* and *E. multilocularis*. Previous studies have reported a high rate of CE in livestock and humans in this province [32]. Consequently, Razavi Khorasan should be considered an endemic area for both *E. granulosus* and *E. multilocularis* infection. Razavi Khorasan Province is a tourist area, and many travelers are at the risk of exposure to these zoonotic diseases. The province has extensive agriculture and export of fruits to other parts of the country. For these reasons and because of close contact of humans with infected domestic dogs and other definitive hosts that forage for food on farms and gardens of this region, it is important to initiate intensive health initiatives.

In conclusion, our study confirms the presence of the life cycle of *E. multilocularis* in the Chenaran region of northeastern Iran. In this cycle the red fox, jackal, wolf, hyena, and dog play the role of the definitive host, and efforts are underway to elucidate the intermediate host of this parasite.

Since the current survey is the first evidence of existence of *E. multilocularis* in domestic and wild animals in northeastern Iran, further studies should be conducted to investigate the presence of *E. multilocularis* in other parts of the country.
